# Quality of Life and Social Life Situation in Islet Transplanted Patients: Time for a Change in Outcome Measures?

**Published:** 2011-08-01

**Authors:** E. Häggström, M. Rehnman, L. Gunningberg

**Affiliations:** 1 *Clinical Student Supervisor, Departments of Transplantation and Surgery, Uppsala University Hospital, Uppsala, Sweden.*; 2 *International Coordinator, Mälardalen University, School of Health, Care and Social Welfare, Eskilstuna, Sweden.*; 3 *Associate Professor, Department of Public Health and Caring Sciences, Section of Caring Sciences, Uppsala University, Uppsala, Adjunct Assistant Professor, School of Nursing, University of California, San Francisco, CA, USA.*

**Keywords:** Islet transplantation, Fear of hypoglycemia, Social life situation, Quality of life, Severe diabetes type 1

## Abstract

Background: One of the overall goals in health care is to prolong life, increase patients’ wellbeing and quality of life. Many of patients with severe insulin-dependent diabetes mellitus experience fear of hypoglycemia (FoH), which forces them to change their lives both physically and socially to avoid episodes of hypoglycemia.

Objective: To investigate the quality of life and the social life situation, with special focus on the consequences of FoH in islet transplanted patients.

Methods: 11 patients (4 women and 7 men) were included; they have undergone islet transplantation at Uppsala University Hospital during the period 2001–2009. Short Form 36 (SF-36) and the Swedish version Hypoglycemia Fear Survey (Swe-HFS) were used to investigate quality of life, in relation to FoH. In addition, telephone interviews were conducted to investigate the patients social life situation in relation to FoH, after islet transplantation and were analyzed using a content analysis method.

Results: The mean value for quality of life was lower than that in the normal population. 3 out of 10 patients experienced FoH; one patient declined to answer the questionnaire. 3 predominant themes were revealed; one theme associated with pre-transplant, was “struggle for control of social life situation” and two themes associated with post-transplant, were “regain power and control of social life situation” and “at peace with the balance between the present and the future.”

Conclusion: The patients experienced improved control over social life situation while quality of life in relation to FoH may have improved following islet transplantation.

## Introduction

Earlier, in the international community of islet transplantation, there was an agreement in that the primary aim of islet transplantation was to achieve insulin independency and secondly to regain hypoglycemia awareness. In April 2008, the National Institute for Health and Clinical Excellence (NICE) outcome measures for islet transplantation in the UK were changed. Today, the NICE outcome measures are prioritized as “resolution of hypoglycemic awareness, stabilization of glycemic control and not insulin dependence” [1]. The findings in this study might suggest arguments to follow NICE in changing the priority order in measure outcomes for islet transplantation.

The criteria to undergo islet transplantation are severe insulin-dependent diabetes mellitus (IDDM) (absolute insulin deficiency), IDDM debut <40 years of age, been diagnosed >5 years and be between 18 and 65 years of age. The patients must have tried several insulin treatment regimes, received close follow-ups’ by a diabetologist and despite this continue to have difficulties keeping their blood glucose levels stable, experience hypoglycemic episodes repeatedly and fully or partly lost the hypoglycemia awareness. Also, good renal function must be preserved. Due to instant blood mediated inflammatory reaction (IBMIR) some islets are lost during the transplantation and therefore the patients are included in a program which offers between three and four transplantations [2-4].

Patients with severe IDDM often have difficulties keeping their blood glucose levels stable due to fluctuation with severe amplitudes in the blood glucose levels. Approximately 25% of these patients lose the ability to perceive episodes of hypoglycemia, hypoglycemia awareness, due to deficiency in the counter active mechanism of blood glucose. Absence of this ability can lead to coma and in worse case cause death [5, 6]. Many of these patients experience fear of hypoglycemia (FoH), which forces them to change their lives both physically and socially to avoid episodes of hypoglycemia [7-10]. 

One of the overall goals in health care is to prolong life, increase patients’ wellbeing and quality of life (QoL) [11]. QoL is a multifaceted and complex concept with various definitions in the different disciplines and scientific areas. There is no single globally accepted definition for QoL, but the World Health Organization (WHO) has defined QoL as; “an individual’s experience of his/her position in life in the context of the culture and the values one lives in and is in relation to one’s own goals and expectations.” Furthermore, QoL is described as a broad concept and is influenced by several factors: the individual’s physical and psychological health, degree of independence, social relationships, beliefs and relationships to important aspects in one’s surroundings [12].

Whether or not QoL can be quantified and if so how is a matter of controversy [13-16]. Measuring the success of medical advances with only biomedical markers and a “find it—fix it” perspective does not provide a complete picture since humans are more complex than this. This is especially the case with chronic and complex diseases as these conditions require patients to adjust on both a social and psychological level [13].

In this study, social life situation is defined as the relation between the patient and persons in his/her surroundings, at home, at work/school and in their leisure time. It involves the skills, strategies and possible support the patient needs to optimally function and master different problems and situations in everyday life [15-17].

Islet transplantation is still an experimental treatment [4] and the population of treated patients is small. Limited research is available on QoL and social life situation in patients with severe IDDM that have undergone islet transplantation [18]. In the care of these patients the following questions have emerged: how do patients experience their QoL? Do the patients experience any FoH? If so, do the patients have any strategies to avoid hypoglycemia? What are the patients’ experiences of their social life situation before islet transplantation in relation to FoH? What are the patients’ experiences of their social life situation after islet transplantation in relation to FoH? Do the patients feel that it was worth going through the islet transplantation? In order to provide good care by giving information, support and follow-up of the islet transplanted patients it is important to carry out empirical research to obtain increased knowledge for the nursing and medical sciences in this field. The objective of this study was to investigate the quality of life and the social life situation, with special focus on the consequences of fear of hypoglycemia, in islet transplanted patients. 

## Materials and Methods

Design

The study design was descriptive and divided in two parts. A cross-sectional, quantitative part with questionnaires and the other with qualitative, semi-structured interviews.

Population

A total study of all patients, *i.e.*, 14 patients, with severe IDDM that had undergone islet transplantation at Uppsala University Hospital during 2001–2009 and who were not previously included in a QoL study. Three patients chose not to participate in this study. Eleven patients—four women and seven men, aged 44–64 years with a mean age of 56.2 years were included. Eleven Short Form-36 (SF-36) and ten Swedish version of Hypoglycemia Fear Survey (Swe-HFS) questionnaires were returned; one patient chose not to answer the Swe-HFS questionnaire due to no longer having diabetes.

Data collection

SF-36 measures health-related of life (HRQL) which deals with the patients’ experiences of the consequences of illness/impairment and physical capacity, vitality and psychological well being. SF-36 is divided into two domains—physical health and psychological health—and includes eight scales: physical function, role-physical, body pain, general health, vitality, social function, role-emotional, mental health. The scales summarize the responses in 35 items, where one item is not included in the scale construction. The items are coded and transformed into scales from 0–100, where ‘0’ indicates poor functioning or impaired QoL whereas ‘100’ indicates good functioning and QoL [19]. 

The Swe-HFS is divided into two sub-scales—a Worry scale and a Behavior/Avoidance scale. Swe-HFS consists of 23 items rated on a 5-point Likert scale. The total score ranges from 0 to 92, where ‘0’ indicates no fear of hypoglycemia and ‘92’ reflects the worst possible fear of hypoglycemia [20].

The telephone interviews were conducted with the aid of an interview guide consisting of seven open questions which were constructed in collaboration with experts in the area. The main questions focused on social life situation in relation to FoH pre- and post-transplantation and if the transplantation was worthwhile. In addition to the original seven questions probes were used to obtain more detailed and richer answers [21]. The interview guide was tested in a pilot interview before the study. No questions in the interview guide were changed.

Procedure

This study was conducted between March and April 2010. The subjects were found by a directory listing of all patients that had undergone islet transplantation at Uppsala University Hospital in Uppsala, Sweden during the years 2001–2009.

A letter of inquiry for participation in the study, information of the aim and content of the study, a written informed consent form, the questionnaires SF-36 and Swe-HFS and a return envelop were sent to the patients. Reminders were sent out to seven patients. 

The interviews were carried out in the order the questionnaires were returned. Interviews were recorded and conducted by the interview guide. One of the authors conducted all the interviews to achieve interviewer consistency in language and technique while transcription and processing of the text was carried out by both authors. 

The study participants were given a specific code attached to a code key to secure patient integrity and confidentiality. All questionnaires, interviews and reply envelopes were marked according to the code key [22, 23]. 

Data analysis

All numeric data from the questionnaires was analyzed by SPSS^®^ for a descriptive summary with mean, median and standard deviation values. The data was subsequently interpreted by means of calculations on the general population for SF-36 and Swe-HFS [19, 20, 24]. The data calculated in this study refers to Swedish general population in the SF-36 questionnaire and to the general population of patients with IDDM in the USA in the Swe-HFS questionnaire (Tables 1 and 2).

**Table 1:** The patients experience of HRQL

	Physical Health				Psychological Health			
	PF^1^	RP^1^	BP^1^	GH^1^	VT^1^	SF^1^	RE^1^	MH^1^
Study group Mean (n=11)	64.0	61.4	63.8	56.6	43.6	69.3	66.7	65.8
Median (n=11)	70	75	62	52	45	62,5	100	72
SD (n=11)	32.5	42.4	20.9	20.6	19.9	28.2	42.2	26.1
General population Mean^2^ (n=8930)	87.9	83.2	74.8	75.8	68.8	88.6	85.7	80.9
SD^2^ (n=8930)	19.6	31.8	26.1	22.2	22.8	20.3	29.2	18.9

1 Physical function (PF), Role function – Physical (RP), Body Pain (BP), General Health (GH), Vitality (VT), Social function (SF), Role function – Psychological (RE) and Mental Health (MH).

2 Reference values for the Swedish general population: SF-36 [19].

**Table 2:** Strategies for behavior and fear of hypoglycemia

	Behavior	Fear	Total
Study group Mean (n=10)	12.5	12.3	24.8
Median (n=10)	11.5	11.5	22
SD (n=10)	7.3	7.7	11.9
General population Mean^1^ (n=108)	28	38	66
SD^1^ (n=108)	5	12	15

1 Reference values for the general population of patients with IDDM in the USA [24].

In the content analysis of the transcribed interviews Granheim and Lundman’s method of meaning unit condensation and categorization were used [25, 26]. Verbatim transcription of an interview is no guaranty for a correct interpretation since vocal language includes emphasis, pauses, variation in word flow, all of which lend different meaning to a text. This has been taken into consideration during the interpretive process to ensure reliability and validity in the data analysis [25].

Ethical considerations

The study was approved by the head of the Surgical Department and followed the principles set out in the Declaration of Helsinki, as well as national and local ethical guidelines for research [22, 23]. Patients received written and verbal information about the study. Participation was voluntary and participants were free to withdraw at any time. All data were treated confidentially. 

## Results

The patients experience of HRQL

In SF-36 the subjects had a lower mean value for HRQL throughout the eight different subscales compared to the general population, which indicates that they experienced a lower level of HRQL compared to what is normal. Within the study-group, the range was relatively large in the eight subscales but the mean value and the median showed that the experienced HRQL still remained above 50. An exception was in the category vitality (VT) where the mean value was 43.6 and the median was 46 (Table 1).

Fear of Hypoglycemia

Swe-HFS showed that most of the patients had little or no FoH. The study group scored 12.5 in behavior, 12.3 in FoH and 24.8 in total, which is a lower and better result than the general population with IDDM (Table 2).

Strategies to avoid hypoglycemia

Swe-HFS showed that five out of ten patients always carried sugar with them. One subject did not carry any sugar. Seven patients controlled their blood glucose level often or always, if they knew they were going to participate in a long meeting or going to a party. However, three patients sometimes or often kept their blood glucose levels higher than recommended when they knew they were attending a long meeting or a party. One subject ate a larger snack before going to bed at night while nine patients never or rarely ate before bedtime. Two patients avoided activity when blood glucose levels were low. Two subjects kept their blood glucose level higher when they were to be alone or avoided being alone at any time. The other patients did not take any prevention in relation to if they were to be alone. 

Experience of social life situation in relation to fear of hypoglycemia pre-transplantation

**Figure 1:**
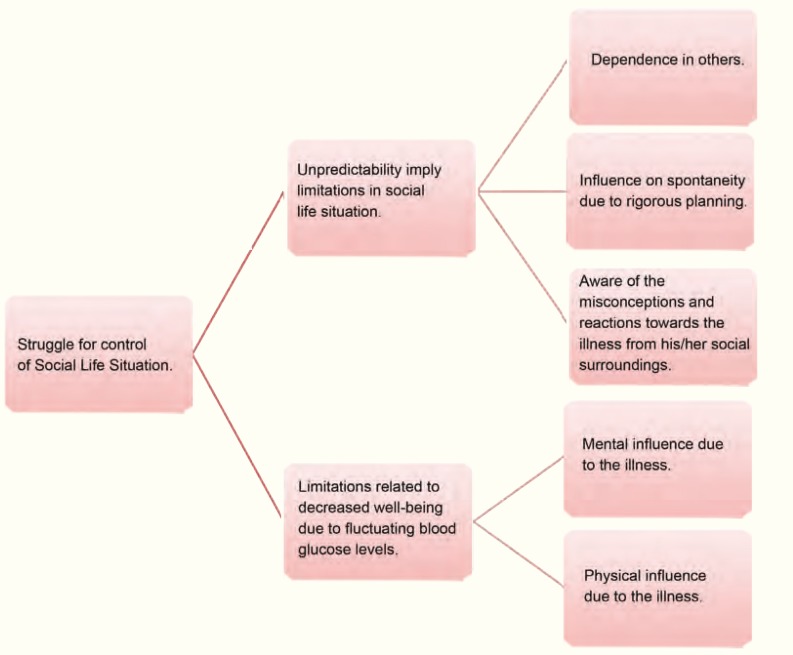
Patients experience of social life situation in relation to fear of hypoglycemia before islet transplantation

One theme identified was *struggle for control over social life situation* as the disease’s unpredictability and diminished state of well being meant limitations. Patients told of different strategies and ways to handle everyday life prior to transplantation. Aspects surfacing in the struggle for control create the subcategories and categories (Fig 1).

Experience of social life situation in relation to fear of hypoglycemia post-transplantation

**Figure 2:**
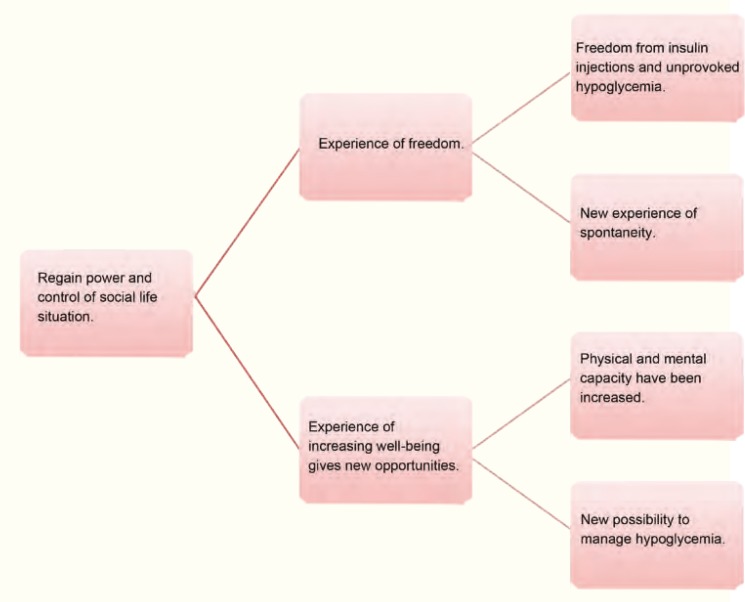
Patients experience of social life situation in relation to fear of hypoglycemia after Islet transplantation

Another theme was *regaining power and control over social life situation* after islet transplantation. The patients experienced feelings of liberation and increasing well being. The subcategories and categories developed from aspects involving regain of control and newly won power over different everyday life situations (Fig 2).

Experience of islet transplantation as worthwhile

**Figure 3:**
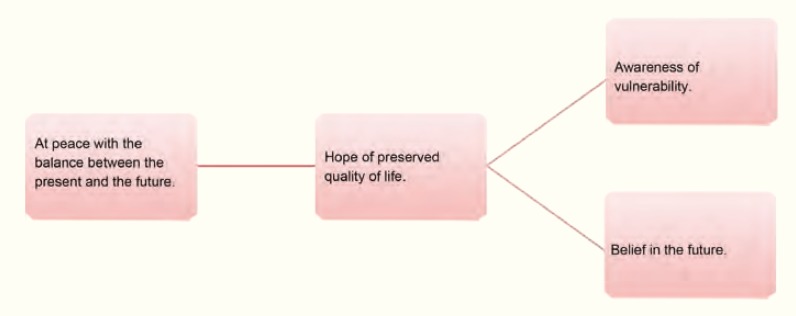
Patients experience if it was worthwhile to go through with the Islet transplantation

One further central theme was, *at peace with the balance between the present and the future*, where aspects such as vulnerability and at the same time faith in the future involve a feeling of hope. The subcategories are created from the stories of possibilities among adversities (Fig 3).

## Discussion

The mean values for HRQL for the study group were lower than that in the general population and is not unexpected [19], but the large range within the group suggests that the study group contains subgroups. Our results reveal that one subgroup includes the patients that have regained insulin independency and hypoglycemia awareness. The other subgroup consists of patients that have regained insulin independency and hypoglycemia awareness but had to return to insulin again. The interviews revealed that despite a decreased level of QoL, it was still kept at a level above that experienced pre-transplant.

Swe-HFS showed that the patients experienced a lower level of FoH than the general population of patients with IDDM [24]. Would a longitude study with measurement pre- and post-transplant show any differences, as Barshes and colleagues’ study or could patient personality traits related to worry have an impact [18]? Most of the patients seldom or never used any strategies to avoid hypoglycemia after islet transplantation. The most common method strategy was measuring the blood glucose level often. Had we seen any differences in strategies to avoid hypoglycemia if measurements had been done pre-transplant?

A significant pattern between pre- and post-transplant social life situation was evident in most of the interviews while the struggle for control was a constant, dynamic and shifting process. Parallels can be drawn to what Parse means is changing and shifting patterns in perspective which weave themselves through life and through the bond between people and their surroundings [14]. The degree of struggle varied widely as some patients succeeded more than others while strategies and resources used in the struggle were very similar. For example, all patients told of some degree of dependence on others, the need for planning and understanding from others as well as the consequences of mental and physical limitations in everyday life. Some patients had a functioning work situation; education and or leisure time both prior to and following transplantation. Others first became successful with these aspects following transplantation. Nevertheless, improvement following transplantation was shared, however sometimes only temporarily.

Social life situation was improved after transplantation for most patients. However, it was noted that in several patients, complex illnesses and related complications influenced the social life situation more than FoH itself. The interviewer encouraged participants to try to disregard those other aspects in order to get a clearer picture of the influence of FoH. This reveals a strong correlation to Hörnqvist’s and Parse’s theories that it is difficult or even impossible to evaluate individual categories in the QoL concept. Interactions between categories make the human unitary and non-dividable. We believe that this aspect in the result is in accordance with Parse’s assertion that QoL cannot be measured with quantitative measures but that QoL reveals itself through the narratives of individuals. Notwithstanding complications, the interviewees expressed that even though there were setbacks, it was still worth going through islet cell transplantation [14, 16].

That last central theme that arose was that all interviewees, regardless of the degree of success in the treatment, told of experience of satisfaction after islet cell transplantation. Most had previously undergone renal transplantation so the guidelines after transplantation were not seen as problematic in the social life situation. Narratives of vulnerability were woven with narratives of faith in the future and developed a concept of hope. Miller defines hope as “… a state of being characterized by an anticipation of a continued good state, an improved state or a release from a perceived entrapment” [27]. Furthermore, hope is described as “…the act by which the temptation to despair is actively overcome.” This act surfaces in the narratives as patients tell of how they chose to live their everyday lives despite awareness that the transplantation is not necessarily a permanent guaranty for health.

Informing and supporting patients in difficult situations is part of the work of caring. To undergo islet transplantation is not entirely free of risks. It involves changes in life style, as lifelong drug treatment and fundamental principals when it comes to hygiene and infections. The end result of islet transplantation is not predictable. There are many aspects to think about before a patient decides to take on the offer of islet transplantation. Up until now, there are very few studies in the area of islet transplantation, QoL and social life situation in relation to FoH. Therefore there is little information available. Our hopes are that the results of this study can be of support in decision making of islet transplantation and give some references to how life as islet transplanted can be. The results of the study are important both clinically, as well as from a nursing and medical scientific perspective. 

Patients who regained insulin independence and hypoglycemia awareness after transplantation, but who had to eventually return to insulin, still kept the level of QoL above that experienced pre-transplantation. These findings are in agreement with re-prioritization in outcome measures in islet transplantation [1]. Therefore, we ask if it is time to follow NICE in changing the priority order in measure outcomes for islet transplantation?

Two validated and reliability controlled questionnaires were chosen. Both SF-36 and Swe-HFS (the original version HFS), have been used in several international studies [19, 20, 24]. The study sample was small and limited the statistical analyses. The result is not possible to generalize to a larger population, but it may indicate how the patients stand in relation to the general population. An ethic dilemma was that the study group was small and many of the patients knew each other. To secure the integrity and confidentiality the patients were given a unique code.

## Conclusion

The patients experienced increased control of their social life situation in relation to FoH after islet transplantation. Consciousness of vulnerability after transplantation and simultaneously a belief in the future lead to an experience of hope. Most patients had little or no FoH after the treatment. As the concept of OoL is very complex and since the clinical situation in several of the patients were complicated it is difficult to generalize about HRQL. The study has contributed to knowledge in social life situation and the experience of FoH for this group but it is uncertain what QoL truly implies for the patients.

Further Research

The results of the study show that the patients gain increased experience of HRQL after undergoing islet transplantation and that most subjects experienced little or no FoH but the range within the group was large. During the process of the study the following questions emerged: what is the significance of the experience of feeling healthy and well or of having a complex clinical situation, to the experience of HRQL and FoH for our study group? Are the patients who experience high HRQL and no FoH the ones who feel healthy and well? Are those who experience low HRQL and high FoH also those with complex clinical situations? Are there any variations between the subjects, if so what are the underlying circumstances? Therefore, further research is needed to ascertain what the already collected data represents in relation to the patients clinical situations.
